# Trends in Medical Training and Leadership at Academic Orthopedic Programs

**DOI:** 10.7759/cureus.29100

**Published:** 2022-09-13

**Authors:** Seleem H Elkadi, Stiles Donaldson, Emily Krisanda, Michael W Kessler

**Affiliations:** 1 Department of Orthopaedic Surgery, Georgetown University School of Medicine, Washington, DC, USA; 2 Department of Orthopaedic Surgery, MedStar Georgetown University Hospital, Washington, DC, USA

**Keywords:** women, residency, ranking, orthopedics, program director, vice-chair, chair

## Abstract

Introduction: When deciding on which programs to rank or fellowships to enter, medical students and residents may assess the program's prestige and specialty training opportunities. This report aimed to analyze the demographics of orthopedic department chairs and program directors (PDs), focusing on the prestige of their orthopedic training and medical school. Secondary data included fellowship, higher-level education, sex, professorship, years of practice, and total published research.

Methods: We used *U.S. News* and *Doximity* to rank 192 medical schools and 200 orthopedic residency programs based on prestige rankings, respectively. We searched for the department chair, vice-chair, and PD via program websites, Council of Orthopaedic Residency Directors (CORD), Orthopedic Residency Information Network (ORIN), personal websites, LinkedIn, and Doximity. Subsequently, we searched for each individual’s demographic information, education and research history, employment history, and medical school attended.

Results: We gathered data on 268 orthopedic surgeons with leadership positions at academic hospitals. Of the 268, 115 were department chairs, 15 were vice-chairs, 126 were PDs, 11 were both the chair and PD, and one was vice-chair and PD. Of the 268 physicians, 244 physicians were male (91.0%), while 22 were female (9.0%). The average residency reputation ranking overall was 59.7 ± 5.7. More specifically, for chairs, the average was 57.0 ± 8.3 (p < 0.005), and for PDs, the average was 63.6 ± 8.0 (p <0.005). There was no significant difference between chairs and PDs (p = 0.26).

Conclusion: Orthopedic leaders were found to have trained at more prestigious programs. This trend could be explained by increased research opportunities at more prestigious programs or programs attempting to increase their own reputation. 9.0% of the leaders identify as female, which is comparable to the 6.5% of practicing female orthopedic surgeons. However, this further demonstrates a need for gender equity in orthopedic surgery. Assessing trends in the training of orthopedic surgeons with leadership positions will allow a better understanding of what programs look for in the hiring process.

## Introduction

Historically, academic leadership positions such as the chair and program director (PD) were devoted to patient care, research, and teaching, but as the medical system evolves, there has been a shift toward administrative tasks [1]. Recent data has shown that department chairs specifically only spend 40 to 45% of their time on clinical activities, leaving 50 to 55% of their time for budgeting, staffing, financial management, negotiations, and contracting [2]. This information begs the question of whether the prestige of chairs’ and PDs' residency training is essential to their selection.

Past literature has analyzed the qualities and traits of orthopedic leadership, but there is minimal published literature analyzing what is academically required to attain one of these leadership positions [2,3]. Bi et al. recently published a paper analyzing demographic information, residency, and fellowship location of department chairs and PDs at academic institutions. While accounting for national subspecialty size, it was found that orthopedic oncology and orthopedic trauma surgeons were overrepresented while reconstructive surgeons were underrepresented amongst department chairs and PDs. Chairs had more publications than PDs and were more likely to be professors, while PDs were more likely to remain in the same program as their residency training [4].

One area of orthopedics that has gained much attention is the lack of females in leadership positions. The American Academy of Orthopaedic Surgeons (AAOS) 2018 census found that self-reported females only made up 5.8% of the AAOS membership [5]. A study in 2021 showed only 2% of department chairs and 11.2% of PDs identified as female, which continues to lag behind the 5.8% found in the AAOS census [4]. However, in 2016, there was only one female department chair, showing that orthopedics is continuing to move towards becoming a more diverse field [6,7].

There is a paucity of literature on whether medical school or residency program reputation influences who is hired to academic orthopedic leadership positions. Literature has shown that medical school ranking plays a role in the orthopedic surgery match, while residency program reputation contributes to fellowship match results [8-10]. We hypothesize that those in academic orthopedic leadership positions attended medical school and orthopedic residency programs with higher reputation rankings.

This report aims to analyze the demographics of orthopedic department chairs and PDs, focusing on the prestige of their orthopedic training and medical school. Secondary data included fellowship, higher-level education, self-reported gender, professorship, years of practice, and total published research.

## Materials and methods

Two hundred Accreditation Council for Graduate Medical Education (ACGME), Doctor of Medicine (MD), and Doctor of Osteopathic Medicine (DO) residency programs located in the United States as of December 2021 were identified. Program websites, CORD (Council of Orthopaedic Residency Directors) Orthopedic Residency Information Network (ORIN), personal websites, LinkedIn, and *Doximity* were searched for the PD, department chair, and vice-chairs and then subsequently searched for demographic information, education, research history, employment history, and medical school attended for each individual. If there was a discrepancy between information on program websites and other sources, data was recorded from the program websites. All data was gathered in March 2022.

Demographic data included the name of the residency program, professorship level, and sex. Professorship levels included professor, associate professor, assistant professor, professor emeritus, and non-professors. Education and research history included program and year of orthopedic internship and residency; name, year, and title of fellowship; the total number of publications, any master's training (MPH {Master of Public Health}, MBA (Masters in Business Administration}, etc.) or a PhD (Doctorate in Philosophy). The number of publications was obtained from the PubMed publications listed on each individual’s *Doximity* account. If that information was not available, the physician was searched on PubMed manually.

*U.S. News* rankings were used to determine the rankings of each medical school, while *Doximity* rankings for reputation and research were used for residency program rankings. The *U.S. News* research ranking is a composite score consisting of a peer assessment score (15%), residency director assessment score (15%), median Medical College Admission Test (MCAT) score (13%), median undergraduate grade point average (GPA) (6%), acceptance rate (1%), faculty resources (10%), total federal research activity (30%), and average federal research activity per faculty member (10%) [11]. *Doximity* calculates the reputation of residency programs by surveying each orthopedic *Doximity* member, verified by board certification, allowing each to nominate five programs while weighting each vote inversely to the size of alumni [12].

Inclusion and exclusion criteria

Inclusion criteria were status as chair, vice-chair, or PD and professor (professor, assistant professor, or associate professor) at their respective MD or DO ACGME accredited orthopedic residency programs. Exclusion criteria were physicians with interim positions, professor emeritus, non-professor or clinical professor, residency programs outside the 50 States of the United States (thus, excluding Puerto Rico), and residency training in a different specialty.

**Figure 1:**
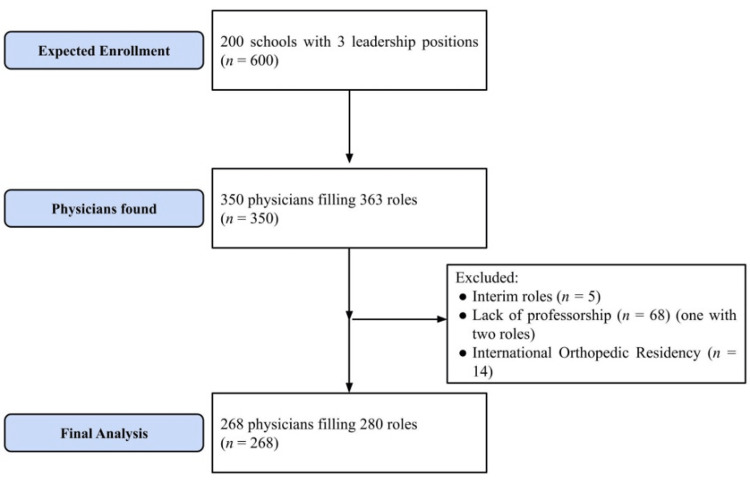
Leadership inclusion and exclusion criteria. Inclusion criteria were set to include physicians with permanent leadership positions with academic titles. Furthermore, to assess residency reputation ranking, only those with residency training inside the United States were included.

Given the 200 ACGME accredited programs and the three positions of leadership we planned to assess, we expected to gather data on 600 physicians. After searching each program's website, we found 350 physicians fulfilling 363 leadership roles. Thirteen physicians held multiple leadership roles in their program. To assess physicians with permanent roles, we excluded physicians with interim roles, which excluded five physicians. To assess physicians with academic titles, we only assessed physicians with professorships in orthopedics at their respective institutions. This excluded 68 physicians, one of which had multiple roles. Finally, we excluded 14 physicians with orthopedic training outside of the United States since those were not ranked in the *Doximity* rankings. This restriction narrowed the total to 268 physicians satisfying 280 roles (Figure 1).

Statistical analysis

Statistical analysis included the assessment of averages and frequencies of demographic and research data. We used univariate data analysis comparing the reputation rankings of orthopedic residency training program rankings of chairs and PDs with two-sample t-tests. In addition, we used univariate data analysis comparing the reputation rankings of orthopedic residency training program rankings of the faculty in leadership positions versus the expected reputation ranking with two-sample t-tests. The expected reputation ranking was calculated by finding the weighted average of the residency ranking. The weighted average was determined first by finding the weighted value of each program, which was obtained by multiplying each residency ranking by the total number of residents at each program. Then, we took the sum of the weighted values and divided this number by the total of 4567 resident positions across the 200 U.S. residency programs to determine the weighted average. To assess for frequency of females in leadership positions, we used a Pearson’s chi-square test using the 2018 AAOS Census as expected data [5]. Finally, to compare fellowship trends in orthopedic academic chairs, vice-chairs, and program directors to orthopedics as a whole, we used the 2018 AAOS Census data for the percentage of each fellowship orthopedic surgeon pursued [5]. P-values less than 0.05 were considered statistically significant. Due to the number of unranked medical schools, statistical analysis was not performed on this data.

## Results

Total leadership statistics

In total, we gathered data on 115 department chairs, 15 vice-chairs, 126 PDs, 11 with both titles of chair and PD, and one with the title of vice-chair and PD. In total, there were 244 males and 24 females (9.0% female). One hundred sixty were professors (59.7%), 62 were associate professors (23.1%), and 46 were assistant professors (17.2%). On average, the physicians completed 23 years of graduating from orthopedic residency. Two hundred twenty-two of the 268 physicians completed one American fellowship (82.8%), 14 had done two American fellowships (5.2%), and one physician did three American fellowships (0.4%). Therefore, 237 of the physicians had completed at least one American fellowship (88.4%) On average, each physician had 60.7 publications on PubMed. Twenty-two of the 268 physicians had master's degrees (8.2%), and four of the physicians had PhD degrees (1.5%), two of which had both a master’s degree and a PhD (0.8%) (Table 1).

**Table 1:** Demographic Data of Academic Orthopedic Leadership. *12 physicians with multiple titles (11 with both department chair and PD, one with vice-chair and PD) SD = Standard deviation, CI = 95% confidence interval, PhD = Doctorate in Philosophy, MD = Doctor of Medicine, DO = Doctor of Osteopathic Medicine

	Department Chair	Vice-chair	Program Directors	Total
Total Number	126	16	138	268*
Males (%)	121 (96.0)	13 (81.3)	122 (88.4)	244 (91.0)
Females (%)	5 (4.0)	3 (18.7)	16 (11.6)	24 (9.0)
Years since Graduating Residency (years) (SD)	28.4 (7.1)	29.7 (12.0)	18.4 (9.0)	23.4 (9.9)
Additional Fellowship (%)	113 (89.6)	15 (93.8)	120 (87.0)	231 (86.2)
1 Fellowship (%)	103 (81.7)	14 (87.5)	116 (84.1)	222 (82.1)
2 Fellowship (%)	9 (7.1)	1 (6.3)	4 (2.9)	14 (5.2)
3 Fellowship (%)	1 (0.8)	0 (0)	0 (0)	1 (0.4)
Number of publications (SD)	91.8 (116.7)	48.7 (31.3)	35.7 (52.1)	60.7 (88.4)
Higher-Level Training (%)	15 (11.9)	4 (25.0)	6 (4.3)	24 (9.0)
Masters (%)	13 (10.3)	3 (18.8)	5 (3.6)	20 (7.5)
PhD (%)	1 (0.8)	0 (0)	1 (0.7)	2 (0.8)
Masters and PhD (%)	1 (0.8)	1 (6.3)	0 (0)	2 (0.8)
MD Degree (%)	124 (98.4)	14 (87.5)	134 (97.1)	262 (97.8)
DO Degree (%)	2 (1.6)	2 (12.5)	4 (2.9)	6 (97.8)
Average Reputation Residency Ranking (CI)	57.0 (8.3)	-	63.6 (8.0)	59.7 (5.7)

**Figure 2:**
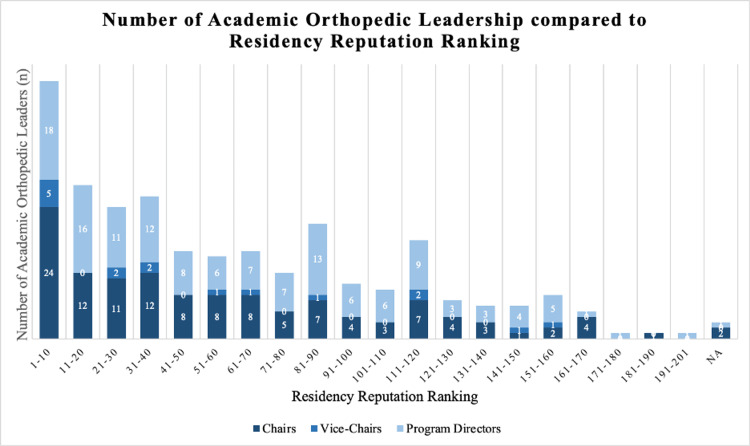
The number of Academic Orthopedic Leadership compared to Residency Reputation Ranking. Reputation rankings were determined using *Doximity*. The programs under NA were former residency programs that have been closed (Letterman Army Medical Center and Fitzsimons Army Medical Center).

**Figure 3:**
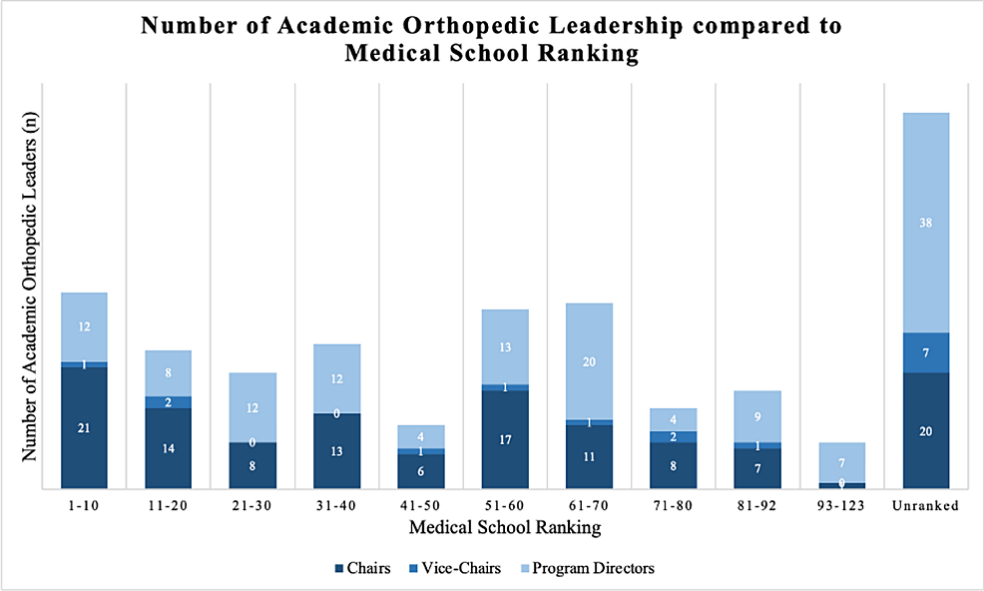
The number of Academic Orthopedic Leadership compared to Medical School Ranking Ranking. Medical rankings were determined using the *U.S. News* research ranking.

Given the number of spots at each program, the expected average reputation ranking was 83.6. The average residency reputation of all the physicians was 59.7 ± 5.7 according to Doximity’s rankings (p < 0.005). The most common residency programs were Hospital for Special Surgery/Cornell Medical Center, Mayo Clinic College of Medicine, and NYU Grossman School of Medicine/NYU Langone Orthopedic Hospital, each producing eight physicians with leadership positions. Thirty-two of the 126 department chairs are chairs in the same program as their residency training (36.6%). The distribution of the programs can be seen in Figure 2. Two hundred and sixty-two physicians are MDs while six are DOs (97.8% MD). The distribution of their medical school ranking according to *U.S. News* can be seen in Figure 3.

Department chair statistics

A total of 126 department chairs were found, with 11 of them also having the title of PD. In total, there were 121 males and 5 females (4.0% female). One hundred eight were professors (85.7%), 11 were associate professors (8.7%), and seven were assistant professors (5.6%). On average, the physicians completed 28 years of graduating from orthopedic residency. One hundred three of the 126 physicians completed one American fellowship (81.7%), nine had completed two American fellowships (7.1%), and one physician completed three American fellowships (0.8%). Therefore, 116 of the physicians completed at least one American fellowship (92.0%). On average, each physician had 91.8 publications on PubMed. Fourteen of the 126 physicians had master's degrees (11.1%), and two had PhD degrees (1.6%), one of which had both a master’s degree and a PhD (0.8%).

Vice-chair statistics

A total of 16 vice-chairs were found, with one of them also having the title of PD. In total there were 13 males and three females (18.8% female). Thirteen were professors (81.3%), two were associate professors (12.5%), and one was an assistant professor (6.3%). On average, the physicians completed 30 years of graduating from orthopedic residency. Fourteen of the 16 physicians completed one American fellowship (87.5%), and one completed two American fellowships (6.3%). Therefore, 15 of the physicians completed at least one American fellowship (93.8%). On average the physicians had 48.7 publications on PubMed. Four of the 16 physicians had master's degrees (25.0%), one of which had both a master’s degree and a PhD (6.3%).

The average residency reputation of all the physicians was 56.6 according to *Doximity’s* rankings. The most common residency program was Wake Forest University School of Medicine. The distribution of the programs can be seen in Figure 2. Seven of the 16 are vice-chair in the same program as their residency training (43.8%). Fourteen of the physicians had their MD while two had a DO (87.5% MD). The distribution of their medical school ranking according to *U.S. News* can be seen in Figure 3.

Program director statistics

A total of 138 PDs were found, with 11 of them also having the title of the chair, and one also having the title of vice-chair. In total, there were 122 males and 16 females (11.6% female). Forty-six were professors (33.3%), 53 were associate professors (38.4%), and 39 were assistant professors (28.3%). On average, the physicians completed 18 years of graduating from orthopedic residency. One hundred sixteen of the 138 physicians completed one American fellowship (84.0%), and four completed two American fellowships (2.9%). Therefore, 120 of the physicians completed at least one American fellowship (87.0%). On average the physicians had 35.7 publications on PubMed. Five of the 138 PDs had master's degrees (3.6%), and one of the PDs had a PhD (0.7%). No PD had both a master's and a PhD. 

Department chairs versus program director residency Doximity reputation ranking

The average residency reputation of all the chairs was 57.0 ± 8.3, according to *Doximity’s* rankings (p < 0.005). The most common residency programs to appear with five chairs each were the University of Rochester and NYU Grossman School of Medicine/NYU Langone Orthopedic Hospital. The distribution of the programs can be seen in Figure 2. Thirty-two of the 126 department chairs are chairs in the same program as their residency training (25.4%). One hundred twenty-four of the physicians had their MD while two had a DO (98.4% MD). The distribution of their medical school ranking according to *U.S. News* can be seen in Figure 3.

The average residency reputation of all the PDs was 63.6 ± 8.0, according to Doximity’s rankings (p < 0.005). The most common residency programs to appear with four PDs each was Hospital for Special Surgery/Cornell Medical Center, Mayo Clinic College of Medicine and Science (Rochester), and SUNY (The State University of New York) Downstate Health Sciences University. The distribution of the programs can be seen in Figure 2. Sixty-three of the 138 department chairs are chairs in the same program as their residency training (58.3%). One hundred thirty-four of the physicians had their MD while four had a DO (97.1% MD). The distribution of their medical school ranking according to *U.S. News* can be seen in Figure 3. We found no statistically significant difference in residency reputation rankings between department chairs and PDs (p = 0.26).

Gender distribution

We performed a Pearson’s chi-squared to assess the distribution of women in leadership. The data obtained from the 2018 AAOS Census was used as the expected value, which stated that women made up 5.8% of orthopedic attendings [5]. Twenty-four women held leadership positions, which was significantly different than the expected 15.5 women (p = 0.031). There was no significant difference in female chairs (p = 0.33), however, there was a significant difference in female PDs (p <0.005), with 16 females as PDs compared to the expected eight females.

When using data that states that females made up 17.8% of females at academic orthopedic institutions as the expected value, the results changed [6]. Twenty-four women overall had a position of leadership compared to the expected 47.7 women (p < 0.005). There were five female chairs compared to the expected 22.4 female chairs (p < 0.005), and 16 female PDs compared to the expected 24.6 female PDs (p = 0.056).

Fellowship data

**Figure 4:**
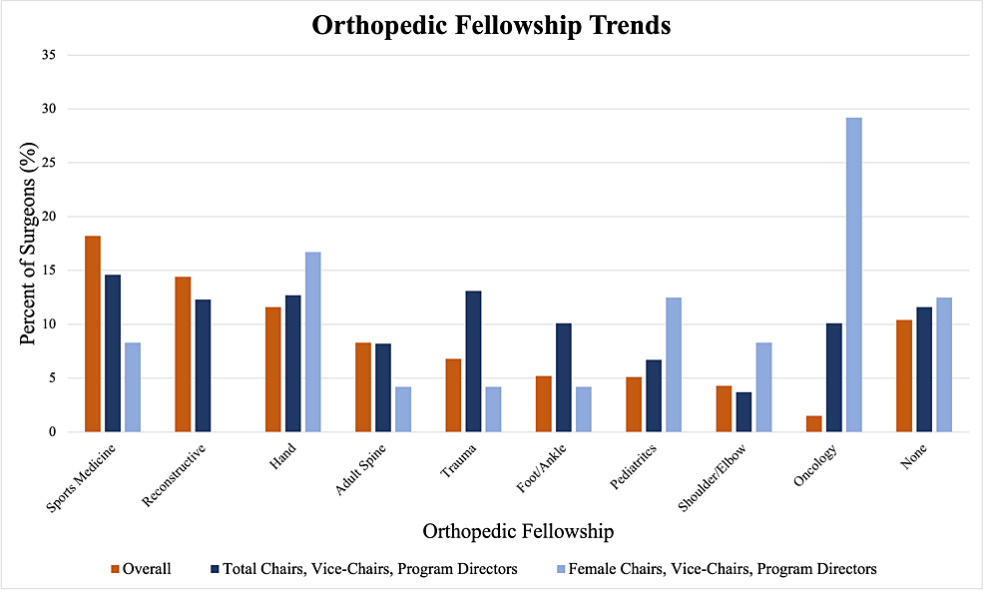
Orthopedic Fellowship Trends The following graph looks at the distribution of orthopedic fellowships in all orthopedics, females in orthopedics, all orthopedic chairs, vice-chairs, and PDs at academic institutions, and female orthopedic chairs, vice-chairs, and PDs at academic institutions. The data for the overall numbers for orthopedics came from the 2018 AAOS census. The data on chairs, vice-chairs, and PDs came from our own data.

We assessed the percentage of each fellowship represented in total and women orthopedic academic chairs, vice-chairs, and program directors. In total, while sports medicine was the most represented (14.6%), oncology and trauma were overrepresented at 10.1% and 13.1%, respectively, compared to the 2018 AAOS census data for all orthopedic surgeons (1.5% and 6.8%, respectively) [5] (Figure 4).

## Discussion

Previous literature has assessed the characteristics and patterns of faculty with leadership positions at academic orthopedic institutions. Common characteristics include significant research history and geographical ties to the location. Furthermore, there has been an increasing trend of faculty with leadership positions who are fellowship trained in either orthopedic trauma or orthopedic oncology. Finally, there remains to be a lack of females in leadership [1,3,4]. Our study aimed to expand on this data and further identify whether the reputation of their orthopedic training program correlates with those in leadership positions.

In order to assess those with leadership positions in academics, we assessed only physicians with professorship (professor, associate professor, or assistant professor) at their own academic institutions who were the chair, vice-chair, or PD. This limitation excluded leaders at hospitals who may interact with residents but do not have an academic title.

We found that around half (136/268) of academic orthopedic leaders were trained at a top 50 residency programs according to *Doximity* reputation rankings, with an average residency program reputation ranking of 59.7. The average residency reputation ranking for department chairs was 57.0, and for PDs was 63.6. There was no significant difference between the reputation rankings for department chairs and PDs, but there was a significant difference in the reputation rankings seen in this study compared to the expected reputation ranking value of 83.6 in all leadership positions.

This data implies that orthopedic leadership often comes from programs with an increased reputation. This trend can be due to a variety of reasons. Often programs with a higher reputation also produce a significant amount of research, which has been shown to be associated with those found in leadership in orthopedics [4]. Also, academic institutions may want to pursue physicians with training at higher ranking residency programs to improve their own reputation further. While many factors go into deciding which programs to rank when matching into a residency program, program reputation may play a role in those hoping to pursue an academic leadership position eventually.

With a large portion of the orthopedic surgeons in academic leadership attending unranked medical schools according to the *U.S. News* ranking, it was difficult to assess the data quantitatively. However, based on the chart, around half (142/268) of physicians came from a top 60 medical school. There are 192 medical schools, which implies a trend for orthopedic academic leaders to attend higher-ranking medical schools [11]. However, we were unable to assess the significance of this trend. Future research should attempt to quantify this data to determine objective trends. 

We found similar statistics in publications, years of experience, and fellowship distribution compared to previous studies [4]. Furthermore, we also found that the orthopedic oncology and orthopedic trauma fellowships were seen in a disproportionately high amount compared to the number of those that pursue these fellowships. We believe this may occur because orthopedic trauma and orthopedic oncology fellowships may result in these physicians remaining within a hospital system rather than in private practice. Chan et al. found that the top two orthopedic subspecialties with job listings, by percentage, in academic centers were orthopedic oncology and orthopedic trauma [13]. Furthermore, it may be easier for orthopedic oncologists and orthopedic trauma surgeons to pursue research and set themselves up for an academic orthopedic leadership position by remaining in a hospital system.

Furthermore, previous literature found that women remain underrepresented in academic orthopedic leadership positions [1,3,4]. Our data continues to support this. At the first glance, using the number of female orthopedic attendings provided by the AAOS, it seems like women are overrepresented in academic orthopedic leadership [5]. However, when using the number of full-time women orthopedic surgery faculty found in academic programs, we found that women continue to be underrepresented in leadership [6]. Correcting this trend may lead to more women pursuing orthopedics out of medical school. A number of studies have shown that women are more likely than men to indicate having a role model or mentor positively influences their pursuit of orthopedic surgery [6,7,14-18]. On the other hand in January 2020, Bi et al. found that 3/153 (2.0%) chairs and 18/161 (11.2%) of PDs were women in all residency programs, while our study found that 5/126 (4.0%) of chairs and 16/138 (11.6%) of PDs with an academic position were women as of March 2022 [4]. This represents a slight increase in women's representation, but it remains disproportionately low compared to the 17.8% of women with positions at academic institutions.

Finally, we assessed orthopedic fellowship trends amongst women. In 2020, Jurenovich et al. surveyed 252 women on their fellowship choices [19]. Women in this survey were more likely to pursue pediatric and hand fellowships and less likely to pursue a reconstructive fellowship compared to the 2018 AAOS census on total fellowship trends amongst all orthopedic surgeons. Similarly, we found that female orthopedic chairs, vice-chairs, and PDs at academic institutions were more likely to have completed a fellowship in pediatrics and hand, but less likely to pursue reconstruction. Women in academic leadership positions were also more likely to have a fellowship in oncology, which aligns with the fact that women tend to choose oncology more often, and oncology-trained orthopedic surgeons are more likely to be chairs, vice-chairs, and PDs (Figure 4). Therefore, even within orthopedics, women are disproportionately represented in each subspeciality compared to their peers. It has been reported that the biggest factor in females producing certain fellowships was pure enjoyment, while mentorship was not found to play a factor in fellowship choice, which contrasts with the importance of having a female model to choose a career in orthopedics [6,7,13-18]. Statistical analysis was not performed due to the limited number of females in academic leadership positions and the fellowship data coming from two different surveys. We recommend that future census collections assess fellowship choice amongst all genders.

Limitations of this study include the lack of standardization and accessibility of data surrounding orthopedic academic leadership, which has been discussed in numerous studies [4,20-22]. Standardization and easy access to this data would allow for a better understanding of trends related to orthopedics. However, cross-referencing *Doximity* with the academic websites allowed us to accurately assess the training programs of all the orthopedic academic leaders. Furthermore, due to the lack of public data surrounding vice-chairs, we could not analyze their training. Whether this data was unable to be found due to a lack of availability or whether programs have opted to have vice-chairs no longer should be assessed.

## Conclusions

In conclusion, this study highlights that the average leader in academic orthopedics trained in a residency program with a higher than average reputation. This trend suggests that these surgeons had more access to research, or could have been hired due to an attempt by programs to improve their own reputation. Finally, women continue to be underrepresented in orthopedic academic leadership, and correcting this could lead to more women pursuing orthopedics.

## Appendices

Table 2 demonstrates the reputation ranking data obtained from *Doxmity*, along with the number of residents at each program and the percentage of residents at this program.

**Table 2:** Doximity’s Orthopedic Surgery Reputation Ranking with Number of Residents

Ranking	School	Number of Residents	Percentage of Residents
1	Hospital for Special Surgery/Cornell Medical Center	45	1.0%
2	Washington University/B-JH/SLCH Consortium	40	0.9%
3	Mayo Clinic College of Medicine and Science (Rochester)	65	1.4%
4	NYU Grossman School of Medicine/NYU Langone Orthopedic Hospital	70	1.5%
5	Duke University Hospital	40	0.9%
6	University of Washington	40	0.9%
7	Massachusetts General Hospital/Brigham and Women's Hospital/Harvard Medical School	60	1.3%
8	Rush University Medical Center	25	0.5%
9	Vanderbilt University Medical Center	25	0.5%
10	UPMC Medical Education	40	0.9%
11	University of Iowa Hospitals and Clinics	30	0.7%
12	Emory University School of Medicine	30	0.7%
13	University of Pennsylvania Health System	40	0.9%
14	University of Utah Health	40	0.9%
15	Carolinas Medical Center	25	0.5%
16	University of California (San Francisco)	35	0.8%
17	Sidney Kimmel Medical College at Thomas Jefferson University/TJUH	30	0.7%
18	Cleveland Clinic Foundation	30	0.7%
19	University of Virginia Medical Center	25	0.5%
20	Johns Hopkins University	30	0.7%
21	New York Presbyterian Hospital (Columbia Campus)	30	0.7%
22	Stanford Health Care-Sponsored Stanford University	35	0.8%
23	University of Texas Health Science Center at Houston	30	0.7%
24	University of Miami/Jackson Health System	35	0.8%
25	University of Minnesota	40	0.9%
26	University of Southern California/LAC+USC Medical Center	40	0.9%
27	McGaw Medical Center of Northwestern University	45	1.0%
28	University of Texas Southwestern Medical Center	30	0.7%
29	University of Michigan Health System	40	0.9%
30	University of Rochester	40	0.9%
31	University of California Davis Health	25	0.5%
32	University of Tennessee/Campbell Clinic	40	0.9%
33	Icahn School of Medicine at Mount Sinai	35	0.8%
34	Prisma Health/University of South Carolina SOM Greenville (Greenville)	20	0.4%
35	Brown University	30	0.7%
36	Case Western Reserve University/University Hospitals Cleveland Medical Center	30	0.7%
37	University of Wisconsin Hospitals and Clinics	30	0.7%
38	Wake Forest University School of Medicine	25	0.5%
39	Mayo Clinic College of Medicine and Science (Arizona)	10	0.2%
40	Western Michigan University Homer Stryker MD School of Medicine	15	0.3%
41	UCLA David Geffen School of Medicine/UCLA Medical Center	30	0.7%
42	University of Maryland	30	0.7%
43	University of South Florida Morsani	20	0.4%
44	Loyola University Medical Center	25	0.5%
45	University of Chicago	25	0.5%
46	Allegheny Health Network Medical Education Consortium (AGH)	25	0.5%
47	MedStar Health/Georgetown University Hospital	20	0.4%
48	University of Colorado	35	0.8%
49	Tufts Medical Center	20	0.4%
50	University of Missouri-Columbia	25	0.5%
51	Montefiore Medical Center/Albert Einstein College of Medicine	30	0.7%
52	University of California (San Diego) Medical Center	25	0.5%
53	Ohio State University Hospital	30	0.7%
54	University of Tennessee College of Medicine at Chattanooga	15	0.3%
55	University of Alabama Medical Center	30	0.7%
56	University of New Mexico School of Medicine	25	0.5%
57	University of Nebraska Medical Center College of Medicine	25	0.5%
58	University of Florida	20	0.4%
59	Texas A&M College of Medicine-Scott and White Medical Center (Temple)	20	0.4%
60	Boston University Medical Center	25	0.5%
61	University of Texas Health Science Center San Antonio Joe and Teresa Lozano Long School of Medicine	35	0.8%
62	Baylor College of Medicine	30	0.7%
63	Prisma Health/University of South Carolina SOM Columbia (Columbia)	20	0.4%
64	Zucker School of Medicine at Hofstra/Northwell at Huntington Hospital	30	0.7%
65	Virginia Commonwealth University Health System	25	0.5%
66	Summa Health System/NEOMED	20	0.4%
67	Tulane University	15	0.3%
68	University of Connecticut	25	0.5%
69	University of Kansas School of Medicine	20	0.4%
70	Zucker School of Medicine at Hofstra/Northwell	20	0.4%
71	Indiana University School of Medicine	30	0.7%
72	Penn State Milton S Hershey Medical Center	25	0.5%
73	Rutgers Health/New Jersey Medical School	30	0.7%
74	University at Buffalo	25	0.5%
75	Yale-New Haven Medical Center	25	0.5%
76	Medical University of South Carolina	20	0.4%
77	Rutgers Health/Robert Wood Johnson Medical School	20	0.4%
78	Oregon Health & Science University	25	0.5%
79	Spectrum Health/Michigan State University	25	0.5%
80	University of Oklahoma Health Sciences Center	30	0.7%
81	Beaumont Health (Royal Oak and Taylor)	40	0.9%
82	MedStar Health/Union Memorial Hospital	10	0.2%
83	University of North Carolina Hospitals	25	0.5%
84	University of Arkansas for Medical Sciences (UAMS) College of Medicine	30	0.7%
85	Mount Carmel Health System	10	0.2%
86	George Washington University	20	0.4%
87	Maimonides Medical Center	15	0.3%
88	Orlando Health	25	0.5%
89	Temple University Hospital	20	0.4%
90	University of Kansas (Wichita)	20	0.4%
91	Cedars-Sinai Medical Center	20	0.4%
92	Akron General Medical Center/NEOMED	15	0.3%
93	University of Louisville School of Medicine	25	0.5%
94	University of Cincinnati Medical Center/College of Medicine	25	0.5%
95	University of Mississippi Medical Center	20	0.4%
96	UMass Chan Medical School	25	0.5%
97	University of Kentucky College of Medicine	25	0.5%
98	Zucker School of Medicine at Hofstra/Northwell at Lenox Hill Hospital	10	0.2%
99	University of Vermont Medical Center	15	0.3%
100	West Virginia University	20	0.4%
101	Henry Ford Hospital	30	0.7%
102	NYU Long Island School of Medicine	15	0.3%
103	Louisiana State University	20	0.4%
104	Naval Medical Center (San Diego)	25	0.5%
105	National Capital Consortium	30	0.7%
106	Los Angeles County-Harbor-UCLA Medical Center	25	0.5%
107	McLaren Health Care/Flint/MSU	15	0.3%
108	University of Michigan Health-West	10	0.2%
109	University of Texas Medical Branch Hospitals	25	0.5%
110	Dartmouth-Hitchcock/Mary Hitchcock Memorial Hospital	20	0.4%
111	Naval Medical Center (Portsmouth)	20	0.4%
112	UPMC Medical Education/Hamot	15	0.3%
113	Geisinger Health System	20	0.4%
114	Univ of North Dakota School of Medicine and Health Sciences	15	0.3%
115	Medical College of Wisconsin Affiliated Hospitals	25	0.5%
116	Kirk Kerkorian School of Medicine at UNLV	20	0.4%
117	Tripler Army Medical Center	15	0.3%
118	SUNY Downstate Health Sciences University	30	0.7%
119	Albert Einstein Healthcare Network	15	0.3%
120	University of Arizona College of Medicine-Tucson	20	0.4%
121	Madigan Army Medical Center	15	0.3%
122	San Antonio Uniformed Services Health Education Consortium (SAUSHEC)	30	0.7%
123	University of Illinois College of Medicine at Chicago	25	0.5%
124	Baylor University Medical Center	15	0.3%
125	St Joseph's University Medical Center	15	0.3%
126	Cleveland Clinic Foundation/South Pointe Hospital	15	0.3%
127	SUNY Upstate Medical University	25	0.5%
128	University of Florida College of Medicine Jacksonville	20	0.4%
129	William Beaumont Army Medical Center/Texas Tech University (El Paso)	25	0.5%
130	University of Arizona College of Medicine-Phoenix	20	0.4%
131	University of California (Irvine)	20	0.4%
132	Methodist Hospital (Houston)	15	0.3%
133	Southern Illinois University	15	0.3%
134	University of California (San Francisco)/Fresno	20	0.4%
135	Detroit Medical Center/Wayne State University	20	0.4%
136	St Louis University School of Medicine	25	0.5%
137	OhioHealth/Doctors Hospital	25	0.5%
138	Wright State University	20	0.4%
139	John Peter Smith Hospital (Tarrant County Hospital District)	30	0.7%
140	St Luke's University Hospital	15	0.3%
141	University of Texas at Austin Dell Medical School	20	0.4%
142	Rutgers Health/Jersey City Medical Center	15	0.3%
143	Howard University	20	0.4%
144	Louisiana State University (Shreveport)	15	0.3%
145	University of Toledo	20	0.4%
146	Marshall University School of Medicine	15	0.3%
147	Ochsner Clinic Foundation	15	0.3%
148	USA Health	15	0.3%
149	University of Puerto Rico*	20	0.2%
150	University of Hawaii	10	0.4%
151	Medical College of Georgia	20	0.3%
152	Broward Health	15	0.5%
153	Stony Brook Medicine/University Hospital	25	0.3%
154	Westchester Medical Center	15	0.4%
155	University of Missouri-Kansas City School of Medicine	20	0.4%
156	WellStar Atlanta Medical Center	20	0.3%
157	Wellspan Health/York Hospital	15	0.4%
158	Texas Tech University Health Sciences Center at Lubbock	20	0.5%
159	Albany Medical Center	25	0.3%
160	St Mary's Hospital and Medical Center	15	0.2%
161	HCA Healthcare/USF Morsani College of Medicine GME: Largo Medical Center	10	0.4%
162	Larkin Community Hospital	20	0.3%
163	Nassau University Medical Center	12	0.3%
164	Kettering Health Network	15	0.4%
165	Community Memorial Health System	20	0.4%
166	McLaren Health Care/Greater Lansing/MSU	20	0.5%
167	Loma Linda University Health Education Consortium	25	0.2%
168	Western Reserve Hospital	10	0.2%
169	OPTI West/Valley Hospital Medical Center	10	0.3%
170	Valley Consortium for Medical Education	15	0.4%
171	Philadelphia College of Osteopathic Medicine	20	0.2%
172	Rutgers Health/Monmouth Medical Center	10	0.2%
173	Cooper Medical School of Rowan University/Cooper University Hospital	10	0.3%
174	Inspira Health Network/Inspira Medical Center Vineland	15	0.4%
175	Kansas City University GME Consortium (KCU-GME Consortium)/HCA Healthcare Kansas City	20	0.3%
176	University of Central Florida/HCA Healthcare GME (Ocala)	15	0.5%
177	RowanSOM/Jefferson Health/Virtua Our Lady of Lourdes Hospital	25	0.5%
178	UPMC Medical Education (Harrisburg)	25	0.2%
179	One Brooklyn Health System/Kingsbrook Jewish Medical Center	10	0.3%
180	Ascension Genesys Hospital	15	0.2%
181	Ascension Macomb-Oakland Hospital	10	0.3%
182	Ascension Providence/MSUCHM	15	0.4%
183	Beaumont Health (Farmington Hills and Dearborn)	20	0.2%
184	Case Western Reserve University/University Hospitals Cleveland Medical Center/Regional	10	0.2%
185	Dwight David Eisenhower Army Medical Center	10	0.2%
186	East Tennessee State University/Quillen College of Medicine	10	0.4%
187	Franciscan Health Olympia Fields	20	0.2%
188	Garden City Hospital	10	0.4%
189	Geisinger Health System (Wilkes Barre)	20	0.2%
190	Henry Ford Macomb Hospital	10	0.3%
191	Jack Hughston Memorial Hospital	15	0.3%
192	Lake Erie College of Osteopathic Medicine	15	0.3%
193	McLaren Health Care/Macomb/MSU	15	0.3%
194	McLaren Health Care/Oakland/MSU	15	0.3%
195	Mercy St Vincent Medical Center	15	0.2%
196	Oklahoma State University Center for Health Sciences	10	0.3%
197	Riverside University Health System	15	0.3%
198	Robert Packer Hospital	15	0.3%
199	Samaritan Health Services	15	0.2%
200	Sinai Hospital of Baltimore	10	0.3%
201	St Elizabeth Youngstown Hospital	15	1.0%

Table 3 demonstrates *U.S. News* rankings used to rank each medical school.

**Table 3:** U.S. News’ Research Rankings for Medical Schools* *Remaining schools were unranked.

Ranking	School
1	Harvard University
2	New York University (Grossman)
3	Duke University
4	Columbia University
4	Stanford University
4	University of California - San Francisco
7	Johns Hopkins University
7	University of Washington
9	University of Pennsylvania (Perelman)
10	Yale University
11	Mayo Clinic School of Medicine (Alix)
11	Washington University in St. Louis
13	University of Pittsburgh
13	Vanderbilt University
15	Northwestern University (Feinberg)
15	University of Michigan - Ann Arbor
17	Icahn School of Medicine at Mount Sinai
17	University of Chicago (Pritzker)
19	Cornell University Weill
19	University of California - San Diego
21	University of California - Los Angeles
22	Baylor College of Medicine
22	Emory University
24	University of North Carolina - Chapel Hill
25	Case Western Reserve University
26	University of Texas Southwestern Medical Center
27	University of Colorado
27	University of Maryland
29	Oregon Health and Science University
29	University of Southern California (Keck)
31	University of Virginia
32	University of Alabama - Birmingham
33	Boston University
33	Ohio State University
33	University of Wisconsin - Madison
36	Brown University (Alpert)
36	University of Florida
36	University of Rochester
39	Albert Einstein College of Medicine
39	University of Iowa (Carver)
41	University of Utah
42	Indiana University - Indianapolis
42	University of Cincinnati
42	University of Minnesota
45	Dartmouth College (Geisel)
45	University of Massachusetts Chan Medical School
45	University of Miami (Miller)
48	University of California - Davis
48	University of California - Irvine
48	University of South Florida
48	Wake Forest University
52	University of Texas Health Science Center San Antonio
53	University of Texas Health Science Center Houston (Mcgovern)
54	University of Nebraska Medical Center
55	Georgetown University
55	Stony Brook University - SUNY
55	Thomas Jefferson University (Kimmel)
55	Tufts University
55	University of Illinois
60	George Washington University
61	Temple University (Katz)
61	University of Connecticut
61	Virginia Commonwealth University
64	Rush University
64	University of Hawaii Manoa (burns)
66	Hofstra University/Northwell Health (Zucker)
66	Rutgers New Jersey Medical School - Newark
66	University of Vermont (Larner)
66	Wayne State University
70	Rutgers Robert Wood Johnson Medical School New Brunswick
70	Saint Louis University
70	University of Arizona - Tucson
70	University of Kentucky
74	University of Oklahoma
75	Augusta University
75	Texas A&M University
75	University of Arkansas for Medical Sciences
75	University of Kansas Medical Center
75	University of Louisville
75	University of Missouri
81	University at Buffalo SUNY (Jacobs)
81	University of New Mexico
83	University of Missouri - Kansas City
83	Virginia Tech Carilion School of Medicine
83	West Virginia University
86	Drexel University
86	University of Central Florida
88	Eastern Virginia Medical School
88	SUNY Upstate Medical University
90	New York Medical College
90	Tripler Army Medical Center
90	University of South Carolina
93-123	Copper Medical School of Rowan University
93-123	East Carolina University (Brody)
93-123	East Tennessee State University (Quilen)
93-123	Edward Via College of Osteopathic Medicine
93-123	Florida Atlantic University (Schmidt)
93-123	Florida International University (Wertheim)
93-123	Florida State University
93-123	Howard University
93-123	Lake Erie College of Osteopathic Medicine
93-123	Lincoln Memorial University (Debusk)
93-123	Louisiana State University Health Sciences Center - Shreveport
93-123	Marshall University (Edwards)
93-123	Midwestern University (Arizona)
93-123	Midwestern University (Illinois)
93-123	Nova Southeastern University Patel College of Osteopathic Medicine (Patel)
93-123	Ohio University
93-123	Oklahoma State University
93-123	Quinnipiac University
93-123	Rowan University School of Osteopathic Medicine
93-123	Texas Tech University Health Sciences Center
93-123	Touro University California
93-123	University of California Riverside
93-123	University of New England
93-123	University of North Texas Health Sciences
93-123	University of Pikeville
93-123	University of Tennessee Health Science Center
93-123	University of Toledo
93-123	Western University of Health Sciences
93-123	West Virginia School of Osteopathic Medicine
93-123	William Carney University College of Osteopathic Medicine
93-123	Wright State University (Boonshoft)
